# A new look at the ventral nerve centre of *Sagitta*: implications for the phylogenetic position of Chaetognatha (arrow worms) and the evolution of the bilaterian nervous system

**DOI:** 10.1186/1742-9994-4-14

**Published:** 2007-05-18

**Authors:** Steffen Harzsch, Carsten HG Müller

**Affiliations:** 1 Max Planck Institute for Chemical Ecology, Department of Evolutionary Neuroethology, Beutenberg Campus, Hans-Knöll-Str. 8, 07745 Jena, Germany; 2 Universität Rostock, Institut für Biowissenschaften, Allgemeine und Spezielle Zoologie, Universitätsplatz 2, 18051 Rostock, Germany

## Abstract

**Background:**

The Chaetognatha (arrow worms) are a group of marine carnivores whose phylogenetic relationships are still vigorously debated. Molecular studies have as yet failed to come up with a stable hypothesis on their phylogenetic position. In a wide range of metazoans, the nervous system has proven to provide a wealth of characters for analysing phylogenetic relationships (neurophylogeny). Therefore, in the present study we explored the structure of the ventral nerve centre ("ventral ganglion") in *Sagitta setosa *with a set of histochemical and immunohistochemical markers.

**Results:**

In specimens that were immunolabeled for acetylated-alpha tubulin the ventral nerve centre appeared to be a condensed continuation of the peripheral intraepidermal nerve plexus. Yet, synapsin immunolocalization showed that the ventral nerve centre is organized into a highly ordered array of ca. 80 serially arranged microcompartments. Immunohistochemistry against RFamide revealed a set of serially arranged individually identifiable neurons in the ventral nerve centre that we charted in detail.

**Conclusion:**

The new information on the structure of the chaetognath nervous system is compared to previous descriptions of the ventral nerve centre which are critically evaluated. Our findings are discussed with regard to the debate on nervous system organisation in the last common bilaterian ancestor and with regard to the phylogenetic affinities of this Chaetognatha. We suggest to place the Chaetognatha within the Protostomia and argue against hypotheses which propose a deuterostome affinity of Chaetognatha or a sister-group relationship to all other Bilateria.

## Background

The Chaetognatha (arrow worms) are bilaterally symmetrical marine carnivores and among the most abundant planktonic organisms. To date, about 120 described species are known worldwide from all vertical ranges of the ocean. Most of them are permanently pelagic but several epibenthic species are also known [1-3]. The chaetognaths range in length from 1–120 mm and are characterized by the presence of horizontally projecting fins and, at the anterior end, two groups of moveable, cuticular grasping spines used in capturing prey. Planktonic specimens are usually glassily transparent. Rapid bursts of swimming caused by dorso-ventral undulations alternate with phases during which the animals lie motionless and sink. Chaetognaths are hermaphroditic and develop directly so that newly hatched larvae display a body organisation that is in many aspects similar to the adult. Their phylogenetic affinities are controversial [4,5]. Chaetognaths have traditionally been placed within the Deuterostomes mainly based on the differentiation of the archenteron seemingly resembling enterocoeli [reviewed in [1,6-8]]. However, Kapp [8] emphasizes the phylogenetically isolated position of the Chaetognatha and designates them as *incertae sedis*. Nielsen [3,9], on the other hand, unites the Chaetognatha together with the Rotifera and Gnathostomulida in the taxon Gnathifera thus suggesting a placement within the Protostomia. Important morphological characters in this debate are e. g. the coelomic epithelia and coelom formation [6,10-12]. Chaetognath cleavage has traditionally been perceived as "radial" and thus as suggesting a deuterostome affinity [discussed in [13]]. However, a recent marking experiment of the first cleavage stages shows a spiral cleavage configuration of the four cell stage thereby suggesting a protostomian relationship [13]. A position within the Protostomia is also supported by ultrastructural features of the brain [14] and by analyses of the genes that code for intermediate filament proteins [15,16].

General information on the chaetognath morphology and anatomy has been summarised in the classical, histological contributions by Hertwig [17], Kuhl [18] and more recently in reviews by Goto and Yoshida [19], Bone and Goto [20], Kapp [1,21], Nielsen [3], and Ax [22]; the most detailed review of their anatomy is probably that of Shinn [2]. Chaetognaths have a complex nervous system that is largely epidermal. The general organisation of their nervous system has been examined by Bone and Pulsford [23], and Goto and Yoshida [[19,24]; reviews [2,20]; see Fig. 2A, B]. More specifically, the fine structure of the brain has received much attention [14,19,25,26]. The layout of the neuromuscular innervation [27] and the ultrastructure of neuromuscular junctions [28] have been described. Much attention has also been focused on sensory organs such as ciliated receptor neurons [23,29-32], the eyes [24,33-35], and conjunction with this also on mechanisms of positive phototactical behaviours [36,37]. Three studies have used immunohistochemical techniques to examine the distribution of serotonin and RFamide-like immunoreactive neurons [27,38] and aspartate immunoreactivity [39] in the central and peripheral parts of the nervous systems.

**Figure 1:**
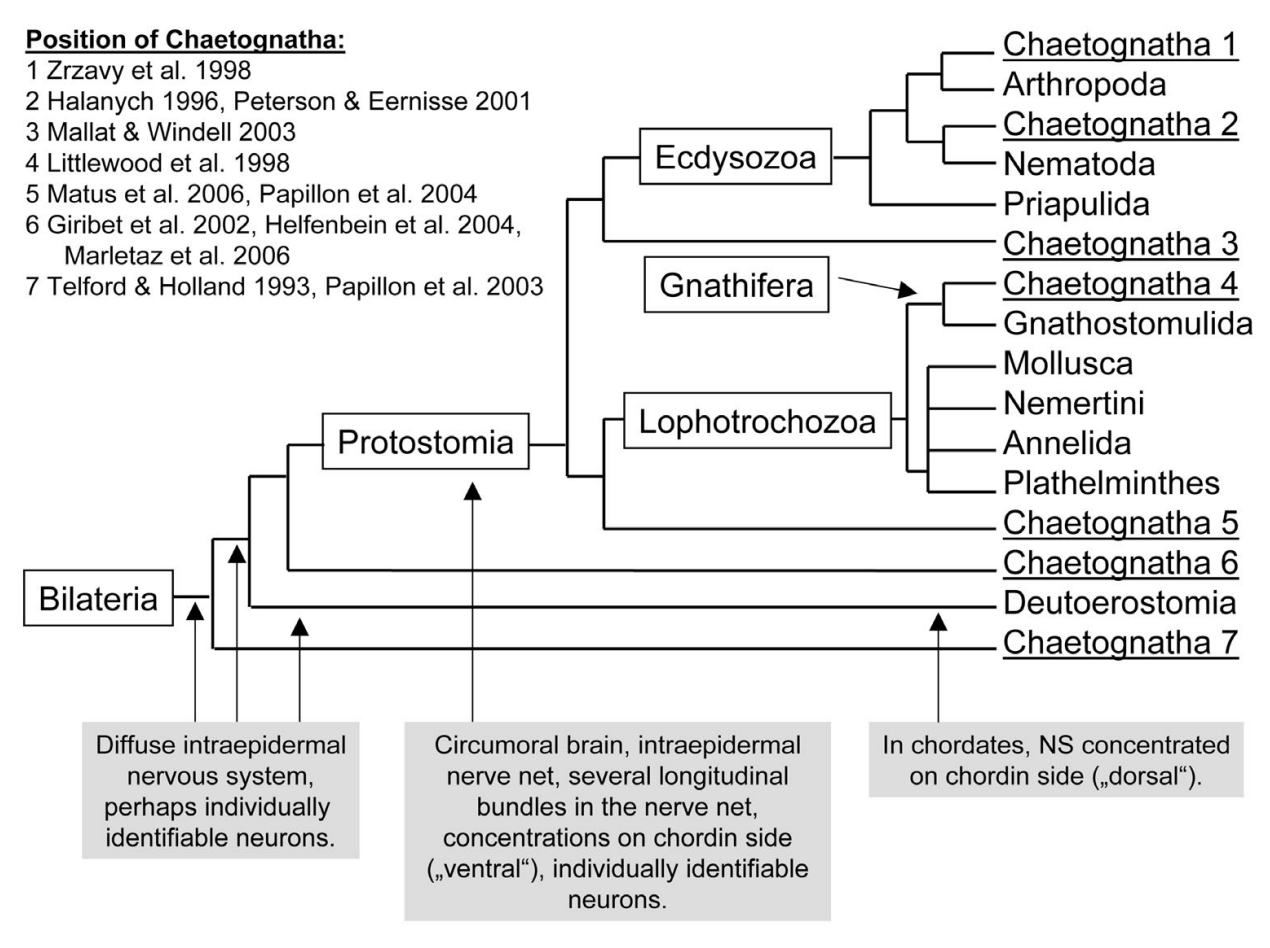
Phylogram of the Bilateria based exclusively on molecular studies to illustrate the competing hypotheses on the position of Chaetognatha ("Chaetognatha 1–7") as suggested by molecular studies. The sources of the hypotheses 1 to 7 are indicated. Boxes indicate some key features of the early bilaterian nervous system (see Discussion for further details).

Systematic analyses of DNA sequences have as yet failed to support an unambiguous hypothesis on chaetognath affinities (summarized in Fig. 1). Wada and Satoh [40] clearly excluded the Chaetognatha from the Deuterostomia in an analysis based on 18S rDNA sequence. In a study also based on 18S rDNA, Telford and Holland [41] proposed a most likely position of the chaetognaths as descendants from an early metazoan branch possibly originating prior to the radiation of the major coelomate groups. Halanych [42] (18S rDNA) suggested a chaetognath-nematode relationship but did not include Nematomorpha and Gastrotricha into his analysis. He recognised long-branch attraction as a possible source of error but tried to minimise this error by a four-taxon analysis. Telford and Holland [43] reported the unusual finding of two distinct classes of 28S rDNA in chaetognaths both of which diverge strongly from other Metazoa. Based on 18S rRNA sequence analysis, Littlewood et al. [44] suggested a sister-group relationship of Chaetognatha and Gnathostomulida. They also proposed the Nematoda as the adelpho taxon to Chaetognatha + Gnathostomulida. Zrzavý et al. al. [45] combined morphological and 18S rDNA sets in a cladistic analysis which suggested a grouping of Onychophora + (Tardigrada + Arthropoda) to be the sister group of chaetognaths. Giribet et al. [46] also combined 18S rDNA data of 145 terminal taxa with 276 morphological characters in a total evidence regime. This analysis yielded a sister-group relationship of Chaetognatha with Nemertodermatida that the authors qualified as unstable and difficult to justify on the basis of morphological/anatomical characters. Peterson and Eernisse [47] analysed with maximum parsimony 138 morphological characters from 40 metazoan groups and 304 18S rDNA sequences (Fig. 8). Their analyses placed the Chaetognatha within the Ecdysozoa and here within the Nematoida similar to the result of Halanych [42]. However, this position was unstable so that the authors "suspect that this placement is potentially artifactual, and are unaware of any morphological synapomorphies shared exclusively by chaetognaths and either nematodes or nematomorphs." They conclude: "Given that chaetognaths have other bilaterian plesiomorphies not found in other ecdysozoans, we suggest that they are more likely basal to the other ecdysozoan clades". Mallatt and Winchell [48] combined large-subunit and small-subunit rRNA sequences. Their chaetognath sequence associated with that of an onychophoran, but this was considered by the authors as unstable and probably due to long-branch attraction. Papillon et al. [49] isolated six *Hox *genes from a chaetognath one of which possessed a mosaic Homeodomain sequence, which in the author's view provided evidence that the Chaetognatha could be an early off-shoot of the triploblastic lineage that predates the deuterostome/protostome split. Helfenbein et al. [50] analysed the complete mitochondrial genome of a representative of the Chaetognatha, *Paraspadella gotoi *(Spadellidae). Their analysis showed that the organisation of the genes in the mtDNA is distinctive among metazoan mtDNAs and that chaetognaths miss many of the mtDNA genes commonly found in other Metazoa. Comparisons of amino acid sequences from mitochondrially encoded proteins in their analysis yielded one single most parsimonious tree that suggests a position of the Chaetognatha as a sister group to the Protostomia [50]. Papillon et al. [51] also analysed the mitochondrial genome of a member of the Spadellidae (*Spadella cephaloptera*). Contrary to the analysis of Helfenbein et al. [51], they placed the arrow worms within the Protostomia and assigned them to the lophotrochozoan clade. Two new papers complete this highly divergent picture. Matus et al. [52] in a Bayesian inference and maximum likelihood analysis of a 56 taxon metazoan tropomyosin data set and 72 genes from ESTs suggest a sister-group relationship of Chaetognatha to the Lophotrochozoa whereas Marlétaz et al. [53] in a maximum likelihood and bayesian inference tree based on the analysis of a concentrated 79 proteins and 11,667 positions ribosomal protein data set proposed a sister-group relationship of Chaetognatha to all Protostomia.

**Figure 2:**
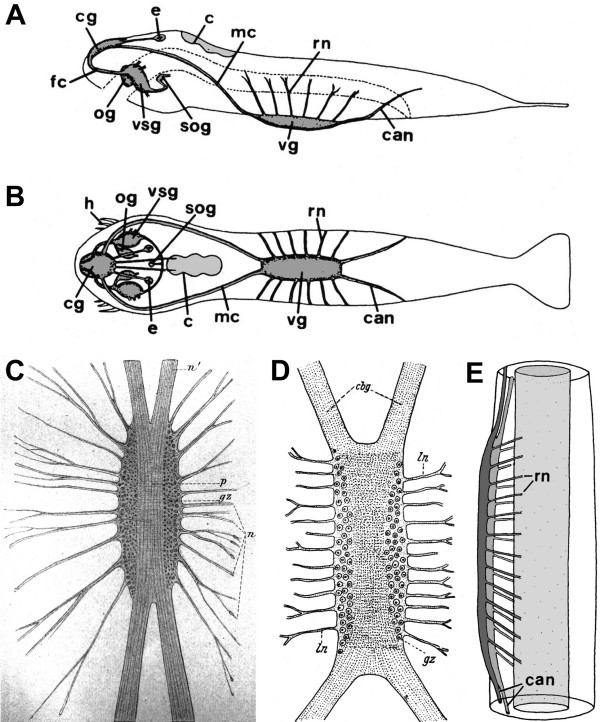
A historic perspective on the nervous system in representatives of the genus *Sagitta*. **A, B: **Schematic representation of the central nervous system in *Sagitta crassa *in a lateral (A) and dorsal (B) view (anterior is to the left; reprinted with permission from Goto and Yoshida 1987). Original abbreviations: C corona ciliata, CAN caudal nerves, CG cerebral ganglion, E eye, FC frontal connective, H mouth hooks, MC main connectives, OG oesophageal ganglion, RN radial nerves, SOG suboesophageal ganglion, VG ventral ganglion, VSG vestibular ganglion. **C: **The ventral ganglion (anterior is to the top) in *Sagitta hexaptera *in an original drawing (reprinted from Hertwig [17]). Original abbreviations: GZ ganglion cells ("Ganglienzellen"), N nerve fibres ("Nervenfibrillen"), P neuropil ("nervöse Punktsubstanz"). **D: **semischematic drawing of the ventral ganglion of *Sagitta bipunctata *(taken from Kuhl [18] as redrawn from Burfield [71]). Original abbreviations: CBG main connective ("Hauptkonnektiv"), GZ ganglion cells ("Ganglienzellen"), LN lateral nerve tracts ("Lateralnerven"). **E: **Generalized scheme of the ventral ganglion of the genus *Sagitta*, lateral view, anterior is towards the top (modified from Shinn [2]). Original abbreviations: CAN caudal nerves, RN radial nerves.

Clearly, the phylogenetic position of arrow worms is unstable in both morphological and molecular studies. Yet, the recent molecular studies on mitochondrial genomes and ESTs seem to favor a position within or close to the Protostomia. Structure and development of the nervous system have always provided strong and important arguments in the discussion on metazoan phylogeny ("neurophylogeny") [54]. Recent examples are the evolution from nerve nets to more centralised nervous systems [55,56], the impact of apical organs, ciliary bands and serotonergic neurons on our understanding of bilaterian evolution [57-59], the fundamental phylogenetic relationships within the Arthropoda [60-62], a possible dorsoventral axis inversion during evolution towards the vertebrate CNS [55,63,64], or the structure of the ancestral bilaterian and chordate brain [65-69]. In the light of the conflicting hypotheses on chaetognath phylogeny, the current paper sets out to give a brief overview over the previous knowledge of the structure of the chaetognath nervous system and to explore the structure of the ventral ganglion in more detail than has been available so far. One main goal of our study was to add a new set of neuroanatomical characters to the discussion on the phylogenetic position of the Chaetognatha. Furthermore, with these data we want to contribute to the debate of how the nervous system of the last common ancester of the Bilateria may have looked like [56,64,68,70].

## Results

### Structure of the chaetognath central nervous system: current knowledge

A brief description of the layout of the adult arrow worm central nervous system will serve as the basis for illustrating our own results. It consists of six ganglia in the head, one unpaired ventral ganglion in the body, nerve tracts connecting these ganglia and peripheral nerves passing out of these ganglia [2,19,20] (Fig. 2). The ganglia in the head are the cerebral ganglion (the brain), a pair of vestibular ganglia, a pair of oesophageal ganglia, and a suboesophageal ganglion (Fig. 2A, B). The brain is located immediately below the surface epithelium of the head and consists of a neuropil core with numerous synapses surrounded by a layer of neuronal cell somata [14,19,20]. Connective nerve tracts are found between the cerebral and the vestibular, the cerebral and the ventral, and the vestibular and the oesophageal ganglia. A commissural nerve bundle behind the oesophagus connects the vestibular ganglia. The anterio-posteriorly running nerves which connect the cerebral and the ventral ganglia are called the main connectives, and those which connect the cerebral and the vestibular ganglia, the frontal connectives [19]. Sensory organs associated with the brain are a pair of eyes [24,33-35], a ciliated loop, the corona ciliata, localised in the dorsal part of the head [2,20,29-32] and the retrocerebral organ, a structure with an unknown putative sensory function [2].

The ventral ganglion is an elongate structure lying between the basement membrane and the epidermis. Two main connectives link it with the brain ganglia and two other nerve tracts continue caudally (Fig. 2A, B) [19,23]. Similar to the brain, the ventral ganglion consists of a central fibrillar neuropil core, flanked by lateral clusters of cell bodies [2,19,20]. Along the length of the ventral ganglion a series of smaller nerves pass out radially (Fig. 2A, B) which branch in the periphery and form a dense ramifying plexus just external to the basement membrane to provide motor innervation to the body musculature and to innervate the ciliary fence receptors [23]. The ventral ganglion controls swimming by initiating contractions of the body wall musculature and co-ordinating mechanosensory input from the numerous ciliary fence receptors in the epidermis [2,20,23]. The available descriptions of the lateral nerves that exit the ventral ganglion in closely related species of the genus *Sagitta *display a remarkable degree of variability over the last 125 years. Hertwig [17] described an irregular array of nerve fibres ("Nervenfibrillen") to emerge from the ventral ganglion (Fig. 2C). Kuhl [18], presenting a modified drawing from Burfield [71], depicts 12 distinct, stout nerve trunks to exit the ganglion on both sides (Fig. 2D). Yet, Goto and Yoshida [19] draw only six radial nerves on each side (Fig. 2B). In Shin [2], again, 12 bilaterally arranged radial nerves exit the ganglion on both sides in a nicely ordered way, much like the arrangement of the segmental nerves in the ventral nerve cord of an annelid or arthropod (Fig. 2E). In the same year, Duvert et al. [39] published a report in which they described 20 – 30 irregularly arranged aspartate-immunoreactive fibre bundles that pass out radially from the ventral ganglion on both sides and spread out diffusely and branch in the periphery, similar to the description already provided by Hertwig [17].

### The structure of the ventral ganglion as revealed by the histochemical localization of actin and a nuclear marker

**Figure 3:**
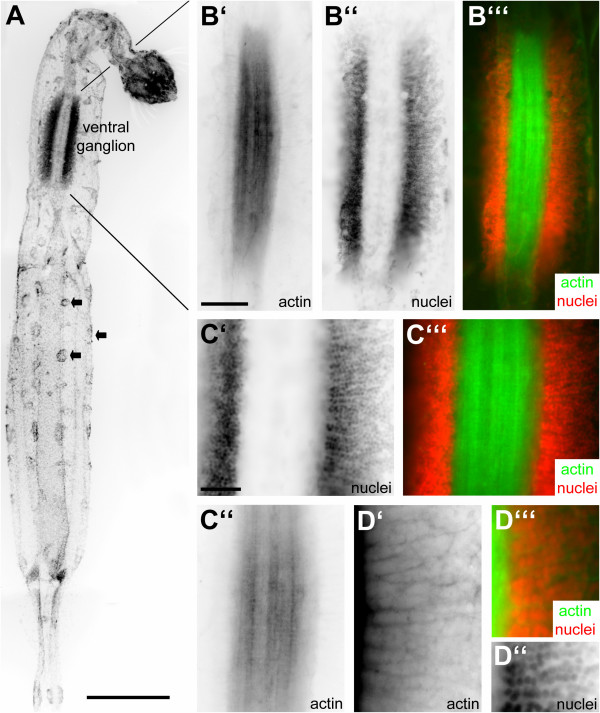
Whole mounts of subadult specimens of *Sagitta *sp. labelled with a nuclear marker (bisbenzimide; red channel) and a histochemical reagent to label actin (phalloidin; green chanel). The images A, B', B", C', C", D', D" are black-white inverted micrographs of specimens labelled with fluoresent reagents. **A: **overview of an entire specimen (nuclear marker) to show the localization of the ventral nerve centre. Arrows identify the cell clusters of fence receptor organs. Scale bar: 500 μm. **B: **higher magnification of the ventral nerve centre (ventral view) labelled for actin (B') to show the central neuropil core and labelled for nuclei (B") to show the flanking zones of neuronal somata. The overlay is shown in B"'. **C, D: **higher magnifications (same set of markers) to show details of the neuropil and arrangement of somata (ventral view). The nerve centre cell somata (C', D") appear to be arranged in rows between the emerging fibre bundles (D') that target the neuropil core in a right angle. Scale bars in B,C: 50 μm.

In whole mounts of juvenile *Sagitta *sp. that were processed with the nuclear marker bisbenzimide the general arrangement of the ventral ganglion is visible (Fig. 3A). Scattered concentrations of nuclei on the body surface mark the location of ciliary fence receptors (arrows in Fig. 3A). Double labelling with bisbenzimide and phallotoxins to visualize actin shows that the ventral ganglion is composed of a central neuropil core that has a rectangular, elongated shape and contains longitudinal fibre tracts (Fig. 3B, C). The neuropil core is on both sides flanked by coherent zones of small (ca. 10 μm) neuronal cell somata (ganglion cells; Fig. 3B, C). Most of these somata are restricted to the sides of the ganglion rather than being located dorsally or ventrally to the neuropil. Fibre bundles that emerge from the cells in the lateral clusters approach the neuropil core from a lateral direction (Fig. 3D'). The ganglion cell somata appear to be arranged in rows between those fibre bundles (Fig. 3D"; and Fig. 6C). At the lateral margins of the soma clusters, large cell bodies are interspersed between the smaller ganglion cells at regular intervals (asterisks in Fig. 6D). These large ganglion cells were already recognized by Hertwig ("große Ganglienzellen" [17]) and also in later studies using methylene blue staining techniques [23,27].

### Tubulin labelling: the ventral ganglion as a condensation of the intraepidermal nerve plexus

**Figure 4:**
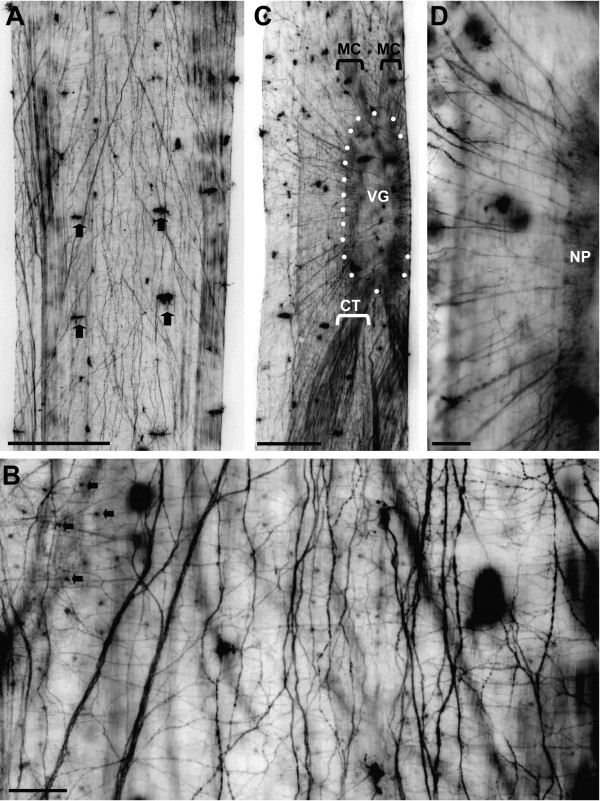
Whole mounts of adult *Sagitta setosa *labelled for acetylated alpha-tubulin (all imaged are black-white inverted). **A: **low-power dorsal view of the trunk surface (slightly posterior to the level of the ventral nerve centre) to show the intraepidermal nerve plexus. Arrows identify ciliary fence receptor organs. Scale bar: 250 μm **B: **higher magnification of the intraepidermal plexus. Arrows identify concentrations of labelled material that are the origin or target of very fine fibres and may correspond to peripheral multipolar neurons. Scale bar: 30 μm **C, D: **An irregular array of ca. 20 – 30 tubulin-labelled fibres and fibre bundles emerges laterally from both sides of the ventral nerve centre as a major source of the peripheral nerve plexus. Abbreviations: CT caudal tracts, MC main connective, NP neuropil, VG ventral nerve centre. Scale bars: 200 μm (C) and 60 μm.

Immunolocalization of acetylated-alpha tubulin in adult *Sagitta setosa *visualized the extensive intraepidermal nerve plexus that extends throughout the entire epidermal surface of the animals (Fig. 4A, B). This dense network of fine nerve fibres is embedded between the basement membrane and the outer epithelial layer [20,27,39] and is known to be involved in the control of muscle contractions [23,28,72]. The network consists of fibres of various sizes many of which extend in a roughly anterior-posterior direction but many transverse fibres are also present (Fig. 4B). Some of the fibres have a beaded appearance suggesting the presence of synaptic varicosities. Our preparations show that the ciliary fence receptors which are also strongly immunolabelled are innervated by tiny branches of the fibre network (arrows in Fig. 4A) suggesting a sensory function of the intraepidermal plexus in addition to the known role in motor control. Hertwig [17] and Bone and Goto [20] observed numerous multipolar neurons associated with the fibres of the intraepidermal plexus. Tubulin immunolocalization revealed conspicuous small concentrations of labelled material (arrows in Fig. 4B) that may correspond to these peripheral neurons but we failed to establish the identity of these concentrations with sufficient certainty.

An irregular array of ca. 20 – 30 tubulin-labelled fibres and fibre bundles emerges laterally from both sides of the ventral ganglion (Fig. 4C, D). These bundles contribute to and are confluent with the peripheral nerve plexus. We did not notice a regularly ordered spacing or distinct bilaterally symmetrical arrangement of these radial nerve bundles as earlier reports had indicated [2,19]. Rather, our findings resemble the pattern of aspartate-immunoreactive fibre bundles that pass out from the ventral ganglion as described by Duvert et al. [39]. Caudally, two thick bundles of tubulin-labelled fibres emerge from the ganglion to connect it to the intraepidermal nerve plexus in the posterior part of the animal (Fig. 4C). These caudal bundles are superficially similar to the radial fibre bundles, yet they contain more closely packed neurites. In summary, in tubulin-labelled preparations the ventral ganglion very much appears to be a condensed continuation of the peripheral intraepidermal nerve plexus.

### Synapsin immunoreactivity: the ventral ganglion as a highly ordered structure

**Figure 5:**
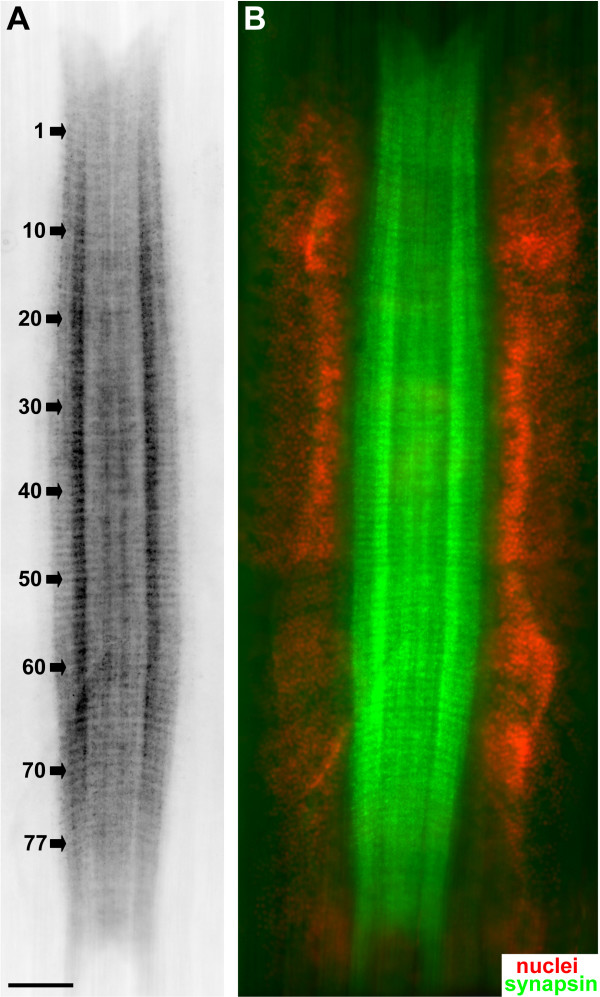
Immunolocalization of synapsin (SYNORF 1; green) in a whole mount of the ventral nerve centre of adult *Sagitta setosa *combined with a nuclear marker (red; ventral views; A is black-white inverted). Synaptic contacts are confined to the central neuropil core which shows a highly ordered subdivision into ca. 80 serially arranged microcompartments. Scale bar: 50 μm.

**Figure 6:**
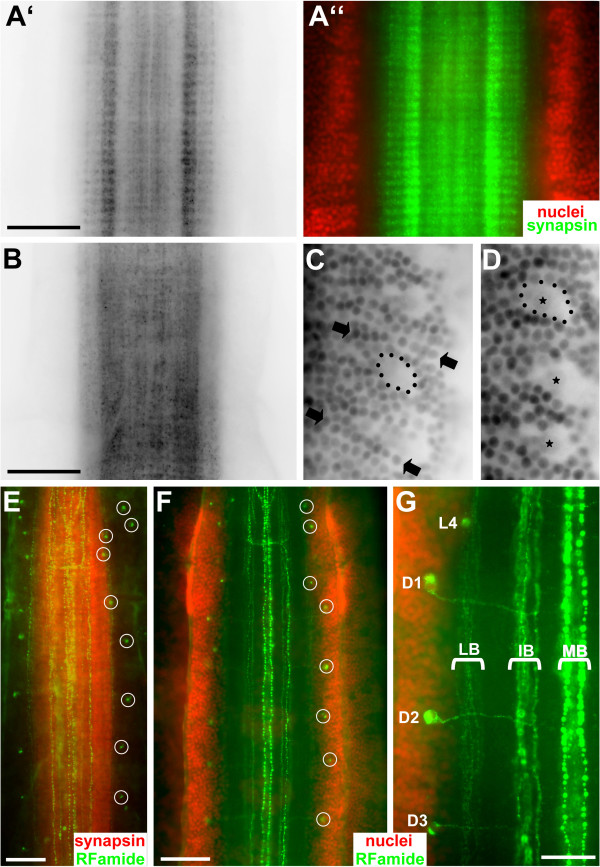
**A, B: **Immunolocalization of synapsin (SYNORF 1; green) in the ventral nerve centre of adult *Sagitta setosa *combined with a nuclear marker (red); higher magnification of the specimen shown in Fig. 5 (A, B are black-white inverted). Overlying the pattern of microcompartments, synapsin immunolabelling is particularly strong in a lateral longitudinal stripe on both sides of the neuropil whereas a narrow medial stripe is devoid of labelling. B shows a slightly more ventral focus level of the specimen shown in A, and the microcompartments are not visible in this focus level. Scale bars: 50 μm **C, D: **peripheral part of the right lateral soma area of the nerve centre (ventral view) labelled with a nuclear dye (images are black-white inverted). The small neurons are arranged in transverse rows (arrows). Note the large cell bodies which are arranged in regular intervals and display only weakly labelled nuclei (asteriks). **E, F, G: **imunolocalization of RFamide (green) in the ventral nerve centre of *Sagitta setosa *(ventral views). Individually identifiable neurons are encircled (E, F) or labelled with letters (G). E is a double-labelled specimen combining RFimmunolcalization with synapsin immunohistochemistry (red). F, G are double-labelled specimen combining RFimmunolcalization with a nuclear marker (red). Abbreviations identifying the longitudinal bundles: IB intermediate bundle, LB lateral bundle, MB medial bundle. Scale bars: 50 μm (E,F), 25 μm (G).

Immunohistochemistry with the SYNORF 1 antibody (Fig. 5) that is directed against presynaptic proteins reveals an overall shape of the synaptic neuropil core in adult *Sagitta setosa *that is similar to that in specimens labelled for actin (Fig. 3B). Clearly, synaptic contacts are confined to the central neuropil whereas the lateral soma regions are devoid of synapses. Synapsin labelling is weak at the anterior and posterior ends of the ventral ganglion suggesting the presence of more fibre tracts than synaptic neuropil in these areas. Interestingly, synapsin immunolocalization reveals a strikingly different picture of the ventral ganglion than tubulin immunohistochemistry ("unorganized nerve plexus") in that it shows a highly ordered subdivision of the neuropil core into serially arranged compartments. The ganglion seems to be composed of an anterior-posterior sequence of ca. 80 transverse microcompartments (Fig. 5, 6A, B). At higher magnification it becomes apparent that, overlying the pattern of microcompartments, synapsin immunolabelling is particularly strong in a lateral longitudinal stripe on both sides of the neuropil whereas a narrow medial longitudinal stripe is devoid of labelling (Fig. 6A, B). Yet, at a slightly more ventral focus level, synapsin immunoreactivity is more evenly distributed across the neuropil core and microcompartments are not visible (Fig. 6B). A comparable serial arrangement of nervous elements in the chaeotognath nervous system so far has been reported in only one other study. Bone and Pulsford [23], using methylene blue and reduced silver staining techniques, provided evidence for the presence of many serially arranged transverse fibres that cross the neuropil core.

### RFamide-like immunoreactivity: individually identifiable neurons

**Figure 7:**
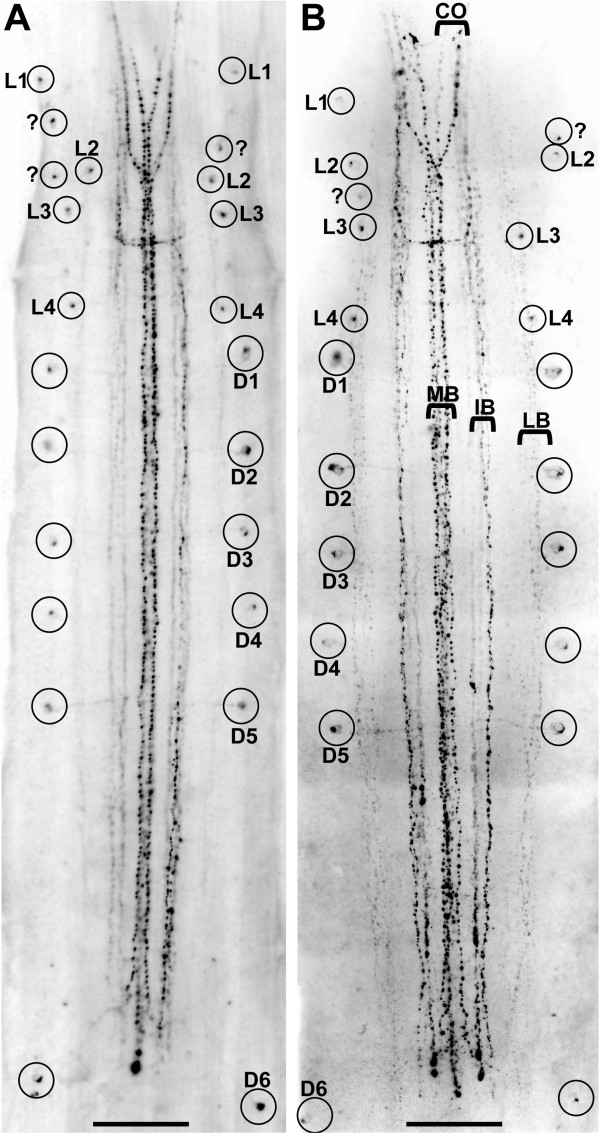
**A, B: **Photomontages of the complete ventral nerve centres (whole mounts, ventral views) of two different specimens of adult *Sagitta setosa*; imunolocalization of RFamide (images are black-white inverted) in the ventral nerve centre (ventral views). Individually identifiable neurons that can be homologized between the two individuals are labelled with letters (for details see text). Abbreviations: CO main connectives, IB intermediate bundle, LB lateral bundle, MB medial bundle. Scale bars: 50 μm.

**Figure 8:**
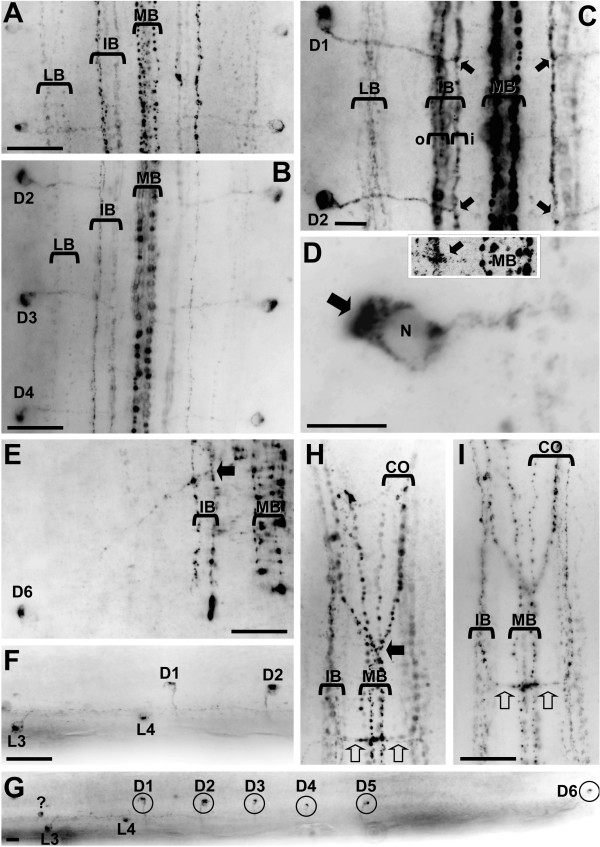
Details of the RFamidergic system in the ventral nerve centre (A-E, H, I are ventral views, F, G are lateral views) of adult *Sagitta setosa *(images are black-white inverted). **A: **Higher magnification of the longitudinal bundles. **B-E: **higher magnification of individually identified D neurons (for an overview of their location see Fig. 7). Within the cell somata, the immunolabelled material is typically concentrated in a cap of cytoplasm opposite to the point of exit of the single neurite (arrow in D). The neurites of the D neurons exit the somata in a medial direction to enter the neuropil core at a right angle to the longitudinal bundles and join the intermediate bundle (IB). They consistenly contact the inner fibres ("i") of the intermediate bundle (arrows in C, E). The inset in D is a high-magnification laser-scan micrograph of the point (arrow) where a D neurite contacts the intermediate bundle. It is unclear if the neurite of this D neuron proceeds more medially to join the medial bundle. **F, G: **lateral views of the ventral nerve centre, anterior is to the left; letters identify individual neurons. **H, I: **anterior part of the ventral nerve centre to show the main connective (CO); H and I show the same specimen at different focus levels and I is slightly more posterior than H. One of the fibres that enters/exits the nerve centre anteriorly crosses the midline to join the medial bundle (arrow in H). Fibres in the intermediate bundle give rise to a characteristic chiasm in the anterior part of the nerve centre (and open arrows in H, I). Abbreviations: CO main connectives, IB intermediate bundle, (i, o label the inner and outer fibres of the intermediate bundle), LB lateral bundle, MB medial bundle, N nucleus. Scale bars: 5 μm (D), 10 μm (C, G), 25 μm (A-B,E-F,I).

In addition to synapsin labelling, immunohistochemistry against RFamide provides further evidence for serially arranged nervous elements in the ventral ganglion of *Sagitta setosa *(Fig. 6E–G). RFamide immunoreactive neurons previously have been described in *Sagitta setosa *[27] and *Paraspadella gotoi *[38], yet these authors did not map the labelling pattern in greater detail. Our study provides conclusive evidence for the presence of individually identifiable neurons in Chaetognatha that can be homologized between different specimens (Fig. 7). Double labelling that combined RFamide immunolocalization with synapsin immunohistochemistry (Fig. 6E) or a nuclear marker (Fig. 6F, G) showed that within the lateral soma zones the cell bodies of the RFamidergic neurons are located mostly close to the interface between the neuropil core and the soma clusters whereas longitudinal RFamidergic fibre tracts are restricted to the ganglion core (Fig. 6E). In Fig. 7, photomontages of the complete ventral ganglia (whole mounts) of two specimens are shown to illustrate the full extent of the RFamidergic system. The labelling pattern was consistent among the more than 20 specimens that we examined. Anteriorly, typically three fibres are present within both main connectives (CO) that link the ventral ganglion to the brain (Fig. 7, 8H, I). Three main longitudinal tracts of RFamide immunolabelled fibres can be distinguished: the medial bundle (MB), the bilaterally paired intermediate bundles (IB), and the paired lateral bundles (LB; Fig. 6G, 7B, 8A) that run along the lateral borders of the neuropil cores as double labelling with the synapsin marker shows (Fig. 6E). All fibres within these longitudinal tracts have a typical beaded appearance. One of the fibres that enter the ganglion anteriorly crosses the midline close to the entry point of the main connectives to join the medial bundle (solid arrow in Fig. 8H). The second fibre in the main connectives is also associated with the medial bundle whereas the third one joins the intermediate bundle (Fig. 8H, I). In the anterior part of the ganglion, fibres in the intermediate bundle run towards the midline and seem to cross over the median nerve bundle towards the contralateral side (Fig. 7, and open arrows in Fig. 8H, I). In contrast to the observations by Goto et al. [38] on *Paraspadella gotoi*, we did not record any RFamidergic fibres that leave the ventral ganglion posteriorly.

The RFamidergic neurons, all of which are unipolar, can be subdivided into two different populations: a first series of neurons with lateral somata (L1–4; small circles in Fig. 7) in the anterior third of the ganglion and a second series of slightly larger dorsal neurons (D1–5; large circles in Fig. 7) in the posterior two thirds of the ganglion the somata of which are located clearly more dorsally than those of the lateral neurons as is apparent in lateral views of the nervous system (Fig. 8F, G). Within the cell somata, the immunolabelled material is typically concentrated in a cap of cytoplasm opposite to the point of exit of the single neurite (Fig. 6G, 8D). The arrangement of the most anterior lateral neurons displayed some variation between individual specimens and in addition to the neurons L1 and L2 that we identified consistently in all six specimens that we analysed at the single-cell level, additional neurons are present in some specimens (small circles labelled with a question mark in Fig. 7). Contrary to L1 and L2, the neurons L3, L4, and D1–D6 were reliably present in all analysed specimens and hence represent typical examples for individually identifiable, bilateral symmetrically arranged neurons. The neurons D1–D5 have an identical morphology and appear to be serially repeated clones. Their neurites exit the soma in a medial direction to enter the neuropil core at a right angle to the anterior-posterior axis (Fig. 6G, 8B, C). The neurites of cells D1–D5 all cross over the lateral longitudinal bundle to join the intermediate bundle. The intermediate longitudinal bundle is composed of an inner and an outer portion (Fig. 8C). The neurites of cells D1–D5 (and also of D6; Fig. 8E) consistently contact the inner fibres of the intermediate bundle which is another indication of their serial identity (arrows in Fig. 8C). In some specimens, the presence of faintly immunolabelled material suggested the neurites of some D neurons to proceed even more medially to join the medial bundle. Screening these regions with high-magnification laser-scan microscopy did not provide conclusive evidence that this is the case (inset in Fig. 8D, arrow).

## Discussion

### The ventral ganglion is a condensation of the intraepidermal nerve plexus

A conspicuous feature of the chaetognaths is that in addition to the central part of the nervous system, a peripheral part is present which is exclusively intraepidermal [2,19,20,39] and which mediates motor innervation to the body musculature and innervates the ciliary fence receptors [23]. By using anti-aspartate immunohistochemistry, Duvert et al. [39] visualized this extensive intraepidermal nerve plexuses both in the head region and in the trunk. In the trunk, muscle contraction seems to be controlled by this profuse intraepidermal network [23,28,67]. Compared to other Bilateria, an unusual feature of the arrow worm neuromuscular system is that in the trunk axonal varicosities lack specialized junctions and are separated from underlying muscles by a thick connective stratum. Acetylcholine is the major neuromuscular transmitter, which reaches muscle cells in the trunk by diffusion through the intervening body wall extracellular matrix [2,20]. Acetylcholinergic fibres deliver their transmitter by boutons that in the trunk region terminate on the epidermis side of the connective stratum and therefore diffusely bath the muscle cells with acetylcholine [27,39]. It should be noted, however, that in the head, nervous fibres which target oesophageal and somatic head muscles, have conventional nerve endings and neuromuscular junctions that display ultrastructural features similar to classic motor end plates [2,28].

Our experiments on tubulin immunolocalization confirm and extend the observations on the structure of the peripheral plexus and ventral ganglion already published by Hertwig [17]. We showed an irregular array of ca. 20 – 30 fibres and fibre bundles to emerge laterally from both sides of the ventral ganglion. These fibres pass out radially to spread out diffusely and branch in the periphery thus being a major source for the peripheral nerve plexus. Duvert et al. [39] described a similar irregular array of aspartate-immunoreactive fibre bundles to spread out from the ventral ganglion. These findings together contradict earlier reports that in *Sagitta*, six [19] or twelve [2,18], distinct, bilaterally arranged radial nerves exit the ganglion on both sides in a serially ordered way. Our findings therefore defy any similarities of the chaetognath ventral ganglion with the internalized ventral nerve cords of e.g. annelids or arthropods that have segmentally arranged nerves projecting into the periphery. Rather, the arrow worm ventral ganglion appears to be a condensation of the intraepidermal nerve plexus. Hertwig [17] and Bone and Goto [20] observed numerous multipolar neurons to be embedded within the intraepidermal plexus. These neurons in the plexus may be the evolutionary precursors of the lateral clusters of cell bodies that flank the central fibrillar neuropil core of the ventral ganglion. During the emergence of Protostomia, the neurons in the plexus may have aggregated to form the centralized nerve centres that we observe in the Protostomia (Fig. 1).

Considering its role to coordinate sensory input from the fence receptors and efferent control of the trunk muscles for swimming behavior, Bone and Goto [20] proposed the ventral ganglion to be part of the central nervous system. From a histological point of view, the ventral ganglion can be seen as a condensation of the epidermal nerve plexus that displays a high degree of centralization. It represents a centralized, yet peripherally located center for complex sensory-motor integration. It is important to note that the chaetognath ventral ganglion is not internalized such as the subepidermal ganglia of Annelida, Arthropoda and Mollusca but remains in an intraepidermal position. It appears that, compared to other Protostomia, the Chaetognatha, by transforming a diffuse nerve net to a more centralized neuronal structure, followed their own distinct evolutionary pathway to generate a ventral nervous center in the trunk for sensory integration and motor control. Therefore, it may be appropriate to term this structure "ventral nerve centre" in order to stress the difference to ventral ganglia of other Protostomia. We suggest that this centralized nerve centre with its specific architecture and intraepidermal location is an autapomorphy of Chaetognatha.

### The serial organization of the ventral nerve centre

Despite the suggested origin of the ventral nerve centre from the peripheral nerve net its central neuropil core displays a high degree of internal organization. Synapsin immunolocalization revealed a highly ordered system of serially arranged synaptic microcompartments in the ganglion core. This compartmentalization may anatomically be linked to a system of serially arranged transverse fibres that cross the neuropil core as reported by Bone and Pulsford [23]. It does not have any equivalent in the ventral ganglia of other Protostomia [73-78] and can be considered another autapomorphy of the chaetognath ventral nerve centre. Our data on the immunolocalization of the neuropeptide RFamide provide further evidence for serially arranged nervous elements in the ventral nerve centre of *Sagitta setosa *and extend previous reports on the localization of this substance in *Sagitta setosa *[27] and *Paraspadella gotoi *[38]. Most importantly, we provide evidence for serially arranged, individually identifiable neurons that can be homologized between different specimens. Although Goto et al. [38] (their Fig. 1d) did not map the pattern of RFamide-immunoreactive neurons in the ventral nerve centre of *Paraspadella gotoi *in detail, a comparison with our data nevertheless suggests that some of the L and D neurons may be evolutionary conserved across different chaetognath species. Papillon et al. [79] described the median *Hox *gene *SceMed4 *in embryos and early hatchlings of *Spadella cephaloptera*. This gene is expressed in two lateral stripes in the middle of the developing ventral nerve centre. The *SceMed4 *mRNA is localized in the bilateral soma clusters but not in the neuropil. These authors suggested that this gene may contribute to the diversity of neuronal subpopulations and to the establishment of distinct axon projection patterns [79]. It would be interesting to explore the expression of this gene in the organism that we studied, *Sagitta setosa*, to see if the region of expression coincided e.g. with the anterior-posterior transition from the "L" to the "D" type of RFamidergic neurons that we describe in the current study.

Individually identifiable neurons (see [80] for a discussion of this concept) seem to be present in the nervous systems of all major taxa within the Protostomia: e.g. Arthropoda [60,61], Annelida [74,77,78,81-83], Nemathelminthes/Cycloneuralia [84,85], basal Mollusca [73,74,86], Plathelminthes [87-89], and Gnathostomulida [90]. Therefore, we suggest that developmental programmes generating neurons with an individual identity must be present in the ground pattern of the Protostomia. A serial arrangement of individual nerve cells cannot only be found in protostomians with typical segmentation such as annelids and arthropods [60,77,78], but also in unsegmented organisms such as Nematoda [85], Plathelminthes [87-89], Chaetognatha (present report), and in organisms the segmental organization of which is unclear such as basal Mollusca [73,86,91]. Our concepts of segmentation in Protostomia mainly rely on work done in annelids and arthropods (reviews e.g. [92,93]). Budd [92] points out that "because segmentation is an evolutionary feature, it must have been acquired in a series of functional intermediates, and the sudden imposition of eusegmentation on a non-segmental precursor seems highly unlikely." In this view, the organization of individual organ systems such as the central nervous system (but not the entire organism) into serially repeated structures may be the starting point in an evolutionary trajectory from which segmentation as we see it in annelids and arthropods emerged [92].

### Evolution of the bilaterian nervous system and the position of Chaetognatha

An intraepidermal nerve plexus is a prominent feature of many basal deuterostomes [55,68]. It is present e.g. in enteropneusts [94], in urochordates such as tunicates [95], and in the basal chordate *Amphioxus *[96]. An extensive intraepidermal nervous system also characterizes many Protostomia [3,55]. Recent investigations on Annelida [77,78,81] and Onychophora (basal arthropods; Mayer and Harzsch, unpublished data) provide new evidence that, in addition to the internalized parts of the nervous system, the peripheral plexus is a prominent feature in these organisms that seem to have retained more motifs of a flatworm-like orthogonal nervous system than has been perceived before. It has recently been proposed that an epithelial (epidermal) nerve plexus (as is also present in Cnidaria; [97-99]) without concentrations into longitudinal cords characterizes the ground pattern of Bilateria [56,64,65] (Fig. 1). Yet, the presence of at least some individually identifiable neurons in basal deuterostomes such as tunicates (Urochordata; [100-102]), and the lancelet *Amphioxus *(Chordata; [96]) indicates that the potential to establish individual identities of the neurons in the plexus may not only be present in the ground pattern of Protostomia (see above) but may date back to the ground pattern of Bilateria (Fig. 1).

The evolution of the deuterostome nervous system is a field of intense research [56,63-66,95,96,103-107] but it is beyond the scope of this contribution to embark into this issue. For the Protostomia, Nielsen [3,58] suggested that a perioral/circumesophageal brain in addition to the intraepidermal nerve plexus characterizes their ground pattern (Fig. 1). Such a circumoral brain can be recognized in most protostomian groups (e.g. [3] Fig. 12.2 therein; and [61,71]) and is by Nielsen [3,58] considered to be one of the key apomorphies of the Protostomia that may have evolved from a circumoral concentration of nerve cells around the mouth as present in Cnidaria. The brain components in Chaetognatha are also arranged in such a typical circumoral pattern (compare Fig. 2).

## Conclusion

In summary, we suggest that in the ground pattern of Protostomia, the nervous system is characterized by the following features (see Fig. 1):

• An extensive intraepidermal plexus (plesiomorphic)

• The developmental programme to establish individual identities of neurons (plesiomorphic?)

• A circumoral brain ring (apomorphic)

• Most likely several longitudinal fibre tracts that are embedded within the peripheral plexus (apomorphic?)

• Ventral centralizations of the plexus that are linked to the brain by longitudinal tracts (apomorphic; "ventral" being the "chordin" expressing side in Protostomia; Lowe et al. 2006)

We conclude that the nervous system architecture of Chaetognatha including ultrastructural features [14] places them within the Protostomia (see also [3]). Phylogenetic affinities of the Chaetognatha to Deuterostomia in our view can be ruled out as well as molecular hypotheses that suggest a sister-group relationship to all other Bilateria [43,49] and, to date, we consider a sister-group relationship of Chaetognatha and Protostomia [46,50] to be unlikely. Based on the differences of the arrow worm brain to the collar-like shape of the typical cycloneuralian brain in Nematoda, we also believe that it is unlikely that Nematoda are the sister-group of Chaetognatha [42,47]. More detailed analyses of the arrow worm ventral nerve centre and especially the brain including studies on neurogenesis will be necessary to explore any potential affinities of this group to specific taxa within the Protostomia.

## Methods

### Experimental animals

Juvenile specimens of an unidentified species of the genus *Sagitta *were obtained from the coastal waters around the Mediterranean island Ibiza (Spain) in March 2006. A plankton net was towed across the surface waters of the Cala Llenya, Cala Vadella, Penyal de s'Aguila, and the Punta Grassio. Adult specimens of *Sagitta setosa *were obtained during a collection trip to the Biologische Anstalt Helgoland, German Bight  in August 2006. Specimens were obtained by horizontal (surface water samples) as well as by vertical (down to 20 meters depth) plankton hauls with the research vessel "MS Aade".

### Histochemistry and immunohistochemistry

Specimens were fixed overnight at 4°C (or for 4 h, room temperature) in 4% paraformaldehyde (PFA) in phosphate buffer (PB; 0,1 M, pH 7.4). Histochemistry and immunohistochemistry were carried on free-floating whole mounts of adult specimens with fluorochrome-conjugated secondary antibodies using standard protocols. After fixation the tissues were washed in several changes of phosphate buffered saline (PBS) for at least 4 h, preincubated in PBS-TX (1% normal goat serum, 0,3% Triton X-100, 0,05% Na-acide) for 1 h and then incubated overnight in the following histochemical reagents and primary antibodies diluted in PBS-TX (room temperature):

• Phalloidin Alexa 488 (1:50; probe for actin; see e.g. [108]; Molecular Probes, obtained from MoBiTec, Göttingen, Germany)

• anti-acetylated alpha-tubulin from mouse (Sigma 1:100; see e.g. [109]).

• anti-FMRFamide from rabbit (1:1000; Diasorin; see e.g. [110]).

• anti-synapsin SYNORF 1 from mouse (1:10; [111]; antibody kindly provided by Prof. Dr. E. Buchner, Universität Würzburg)

For double labelling, combinations of these antisera were used, or the specimens were stained with the nuclear dye bisbenzimide (0.1%, 15 min. at room temperature; Hoechst H 33258), prior to the antibody incubations. Specimens were then washed for at least 2 h in several changes of PBS and subsequently incubated in secondary antibodies against mouse and rabbit proteins conjugated to the fluorochrome Alexa Fluor 488 (Molecular Probes, obtained by MoBiTec, Göttingen, Germany) for 4 hours. Finally the tissues were washed for at least 2 h in several changes of PBS and mounted in GelMount (Sigma). In control experiments, the omission of primary antibodies resulted in the absence of any neuronal labelling.

### Microscopic analysis

Digital images were obtained with a Zeiss Axioskop fitted with a CCD-1300B digital camera (Vosskühler GmbH) and processed with the Lucia Measurement 5.0 software package (Laboratory Imaging Ltd.). Alternatively, samples were scanned with a Leica TCS SP2 AOBS confocal laser-scanning microscope (Lichtmikroskopiezentrum, Institut für Zellbiologie und Biosystemtechnik, Universität Rostock). Those images are based on stacks of between 15 and 20 optical sections (single images are averages of four laser sweeps) of a z-series taken at intervals of 1 μm.

## Competing interests

The author(s) declare that they have no competing interests.

## Authors' contributions

SH and CHGM together obtained the animals, carried out the immunohistochemical experiments and microscopic analyses, and drafted the manuscript. Both authors read and approved the final manuscript.
